# The unique deep sea—land connection: interactive 3D visualization and molecular phylogeny of *Bathyhedyle boucheti* n. sp. (Bathyhedylidae n. fam.)—the first panpulmonate slug from bathyal zones

**DOI:** 10.7717/peerj.2738

**Published:** 2016-12-06

**Authors:** Timea P. Neusser, Katharina M. Jörger, Eva Lodde-Bensch, Ellen E. Strong, Michael Schrödl

**Affiliations:** 1 Biocenter/Dept. II, Ludwig-Maximilians-Universität München, Planegg-Martinsried, Germany; 2 SNSB, Bavarian State Collection of Zoology, Munich, Germany; 3 Smithonian Institution, National Museum of Natural History, Washington, D.C., USA; 4 Center for Geobiology and Biodiversity Research, Ludwig-Maximilians-Universität München, Munich, Germany

**Keywords:** 3D reconstruction, Acochlidiida, Gastropoda, Microanatomy, Mollusca, Oophagy, Scanning electron microscopy, Systematics, Taxonomy

## Abstract

The deep sea comprises vast unexplored areas and is expected to conceal significant undescribed invertebrate species diversity. Deep waters may act as a refuge for many relictual groups, including elusive and enigmatic higher taxa, but the evolutionary pathways by which colonization of the deep sea has occurred have scarcely been investigated. Sister group relationships between shallow water and deep sea taxa have been documented in several invertebrate groups, but are unknown between amphibious/terrestrial and deep-sea species. Here we describe in full and interactive 3D morphoanatomical detail the new sea slug species *Bathyhedyle boucheti* n. sp., dredged from the continental slope off Mozambique. Molecular and morphological analyses reveal that it represents a novel heterobranch gastropod lineage which we establish as the new family Bathyhedylidae. The family is robustly supported as sister to the recently discovered panpulmonate acochlidian family Aitengidae, which comprises amphibious species living along the sea shore as well as fully terrestrial species. This is the first marine-epibenthic representative among hedylopsacean Acochlidiida, the first record of an acochlidian from deep waters and the first documented panpulmonate deep-sea slug. Considering a marine mesopsammic ancestor, the external morphological features of *Bathyhedyle* n. gen. may be interpreted as independent adaptations to a benthic life style in the deep sea, including the large body size, broad foot and propodial tentacles. Alternatively, the common ancestor of Bathyhedylidae and Aitengidae may have been a macroscopic amphibious or even terrestrial species. We hypothesize that oophagy in the common ancestor of Aitengidae and Bathyhedylidae might explain the impressive ecological and evolutionary flexibility in habitat choice in the Acochlidiida.

## Introduction

Almost two-thirds of the surface of the Earth is covered by the deep sea, much of which is still mysterious and untouched, while the diversity of life that inhabits it remains largely unknown (e.g. Bouchet, 2006; Bouchet et al., 2016; Poore et al., 2015) due to technological and economic challenges in sampling deep waters (e.g. Costello et al., 2010; Gage & Tyler, 1991; Jaume & Duarte, 2006). Vast sediment-covered abyssal plains have been suggested to be inhabited by millions of invertebrate species (e.g. Grassle & Maciolek, 1992; Schüller, Brandt & Ebbe, 2013), but may host a comparably poor gastropod fauna in terms of species richness and abundance (e.g. Jörger et al., 2014b; Schrödl et al., 2011a; Schwabe et al., 2007). In contrast, continental slopes may be species rich with abundant snail and slug species (e.g. Rex et al., 2005) though the soft-bodied, and hence fragile, slugs usually suffer from conventional collection techniques (e.g. trawling/dredging) and are often damaged before reaching the surface. Cold and deep waters are known to harbor several plesiomorphic sea slug clades, all belonging to the Nudipleura (Spanish dancers and relatives), indicative of their role as a refuge for relictual lineages (Schrödl, 2003; Wägele et al., 2008). The pathways for colonization of the deep sea are rather well understood for e.g. crustaceans (e.g. Hall & Thatje, 2009; Hessler & Thistle, 1975; Karanovic & Brandão, 2015; Lins et al., 2012; Raupach et al., 2009) and echinoderms (e.g. Ameziane & Roux, 1997; Benitez Villalobos, Tyler & Young, 2006; Smith & Stockley, 2005; Tyler, Young & Clarke, 2000). However, our knowledge about the time scales or evolutionary pathways involved in deep sea colonization in molluscs is patchy and is particularly limited for gastropods (e.g. Eilertsen & Malaquias, 2015; Kano et al., 2013; Mestre, Thatje & Tyler, 2009; Smith & Thatje, 2012; Thubaut et al., 2013).

Recently, molecular phylogenetics rejected the traditional division of Euthyneura in ‘Opisthobranchia’ and ‘Pulmonata’ (e.g. Klussmann-Kolb et al., 2008) and the former pulmonate, opisthobranch and lower heterobranch species were transferred into the newly established heterobranch Panpulmonata (Jörger et al., 2010; Schrödl et al., 2011b). The latter is a megadiverse clade with approximately 25,000–30,000 species (Mordan & Wade, 2008; Schrödl, 2014) including marine, freshwater and terrestrial slugs and snails. Among the marine representatives, only pyramidellid snails are known from deep waters but no panpulmonate slugs. Members of the panpulmonate Acochlidiida have invaded marine and freshwater environments and even the land with 33 species inhabiting coastal mesopsammic areas, seven benthic living freshwater species and one limnic mesopsammic species from the Caribbean (e.g. Jörger et al., 2014a; Schrödl & Neusser, 2010). The recently discovered family Aitengidae comprises two amphibious (Neusser et al., 2011; Swennen & Buatip, 2009) and one terrestrial species (Kano et al., 2015). Curiously, no member of the Acochlidiida is known thus far from deeper waters; exceptions are the tiny marine mesopsammic *Asperspina* sp. from sediments at 58 m off San Juan Island/USA (Morse, 1994) and *Asperspina loricata* inhabiting a coastal underwater dune near Roscoff, France in 50 m (Swedmark, 1968).

From within an integrative framework, we here present the first panpulmonate deep-sea slug. We formally describe the new and macroscopic sea slug species *Bathyhedyle boucheti* n. sp. as the sole known member of the new family Bathyhedylidae and provide an interactive 3D-model of the anatomy. Topological and microanatomical evidence are used to infer the origin and evolution of the Bathyhedylidae.

## Material and Methods

### Material

During the Mainbaza cruise off Mozambique in the Mozambique Channel, two specimens were collected on board the Spanish R/V Vizconde de Eza in April 2009 by Ph. Bouchet, J. Rosado and E. Strong. Both specimens were collected by an otter trawl on soft bottoms. The first specimen (holotype MNHN IM-2000-27917) was collected at a depth between 261 and 264 m along the transect off the mouth of the Zambeze, station CC3150, 19°31′S, 36°46′E, on 13th April 2009. The holotype was fixed in 95% EtOH for molecular studies. The second specimen (paratype ZSM Mol 20140455) was collected (together with *Argyropeza* Melvill & Standen, 1901) at a depth between 437 and 445 m along the Maputo transect, station CC3173, 25°36′S, 33°17′E, on 17th April 2009. The paratype was relaxed in 7% MgCl_2_ and fixed in 3.5% glutaraldehyde in 0.1M Sorenson’s phosphate buffer for microanatomical studies.

### Embedding and sectioning

The glutaraldehyde-preserved specimen was post-fixed in the laboratory in buffered 1% OsO_4_ for 1.5 h in the dark. Subsequently, the specimen was decalcified in 1% ascorbic acid overnight, dehydrated in a graded series of acetone in distilled water (30%, 50%, 70%, 90% and 100%) and embedded in Epon (Luft, 1961). A series of ribboned serial semithin sections of 1.5 μm thickness was prepared using a diamond knife (Histo Jumbo, Diatome, Biel, Switzerland) and by using contact cement at the lower cutting edge (Ruthensteiner, 2008). Finally, the sections were stained with methylene blue-azure II (Richardson, Jarett & Finke, 1960).

### 3D reconstruction and interactive 3D model

The slides were scanned (.vsi format) with an Olympus® dotSlide microscope using the 10× objective. Scanned images were loaded into the image viewer software OlyVia® (Olympus Soft Imaging Solutions GmbH) and every fourth section was recorded as a digital photograph (.tif). Images were converted to 8 bit grayscale format, contrast enhanced and unsharp masked with standard image editing software. A detailed computer-based 3D reconstruction of all major organ systems was conducted with the software Amira 5.2 (Visualization Sciences Group) following Ruthensteiner (2008).

The interactive 3D model (Fig. S1) was prepared according to Ruthensteiner & Heß (2008), but using 3D tools of Deep Exploration 6.5, Right Hemisphere (trial version) and Adobe Acrobat 9.0 Professional Extended. The settings used follow Neusser, Jörger & Schrödl (2011). The 3D model, accessible by clicking on Fig. S1, provides prefabricated views and permits the selection and rotation of the reconstructed organs.

### Analysis by scanning electron microscopy (SEM)

Specimen MNHN IM-2000-27917 was used for the examination of the radula by SEM. The pharynx was removed carefully from the ventral aspect and macerated in 10% potassium hydroxide solution overnight. Any remaining tissue was manually removed with fine dissection pins. After rinsing with distilled water, the radula was mounted on a stub and sputter-coated with gold for 120 s (SEM-Coating-System; Polaron). The radula was examined with a LEO 1430 VP (Leo Elektronenmikroskopie GmbH, Oberkochen, Germany).

### Molecular data

DNA was extracted from a piece of the foot of specimen MNHN IM-2000-27917 using the NucleoSpin Tissue Kit (Macherey and Nagel) following the manufacturer’s protocol. We amplified mitochondrial 16S rRNA as well as nuclear 28S and 18S rRNA using the primers and protocols listed in Jörger et al. (2010). We were unable to amplify cytochrome oxidase subunit I (COI) with the available standard primers. Successful PCR products were cleaned-up with DNA Clean & Concentrator™ (Zymo Research). Cycle-sequencing and sequencing reactions were conducted by the Sequencing Service of the University of Munich using the PCR primers, Big Dye 3.1 and an ABI capillary sequencer. Sequences were edited with Geneious 7.0.6 and checked for possible contamination using BLAST searches (Altschul et al., 1990) and have been deposited in GenBank (GenBank accession numbers: KX721048–KX721050). Alignments for four markers (18S, 28S, 16S rRNA and COI) were conducted using MUSCLE (Edgar, 2004) and ambiguously aligned regions removed with GBlocks (Talavera & Castresana, 2007). For an initial analysis of the phylogenetic relationships of *Bathyhedyle* n. gen., we analysed a concatenated dataset (four standard markers: 18S, 28S, 16S rRNA and COI) for 78 taxa including all major gastropod groups modified after Stöger et al. (2013) (see Table S1) using RAxML-HPC2 (Stamatakis, 2014) via the Cipres Portal (Miller, Pfeiffer & Schwartz, 2010) under the GTR+G model with 1,000 bootstrap replicates in four partitions corresponding to each molecular marker. For a refined analysis, we compiled a taxon sampling targeted towards panpulmonate Acochlidiida (53 taxa, see Table S2 for details) and performed maximum likelihood analyses in RAxML as described above.

### Nomenclatural acts

This published work and the nomenclatural act it contains have been registered in ZooBank, the proposed online registration system for the International Code of Zoological Nomenclature (ICZN). The ZooBank LSIDs (Life Science Identifiers) can be resolved and the associated information viewed through any standard web browser by appending the LSID to the prefix ‘http://zoobank.org/.’ The LSID for this publication is: urn:lsid:zoobank.org:pub:F1DA5BD1-55D6-4F52-BEB5-446762706A52. The LSID for the new family Bathyhedylidae is: urn:lsid:zoobank.org:act:4AC1FF05-EEEC-423F-A0A9-EB4DA636B219. The LSID for the new genus *Bathyhedyle* is: urn:lsid:zoobank.org:act:67E561CD-6B85-478B-98FB-BAAE49C10DAD. The LSID for the new species *Bathyhedyle boucheti* is: urn:lsid:zoobank.org:act:4B32A0B9-72BF-487C-8471-9C657D8E87A9.

## Results

### Molecular phylogeny

**Figure 1:**
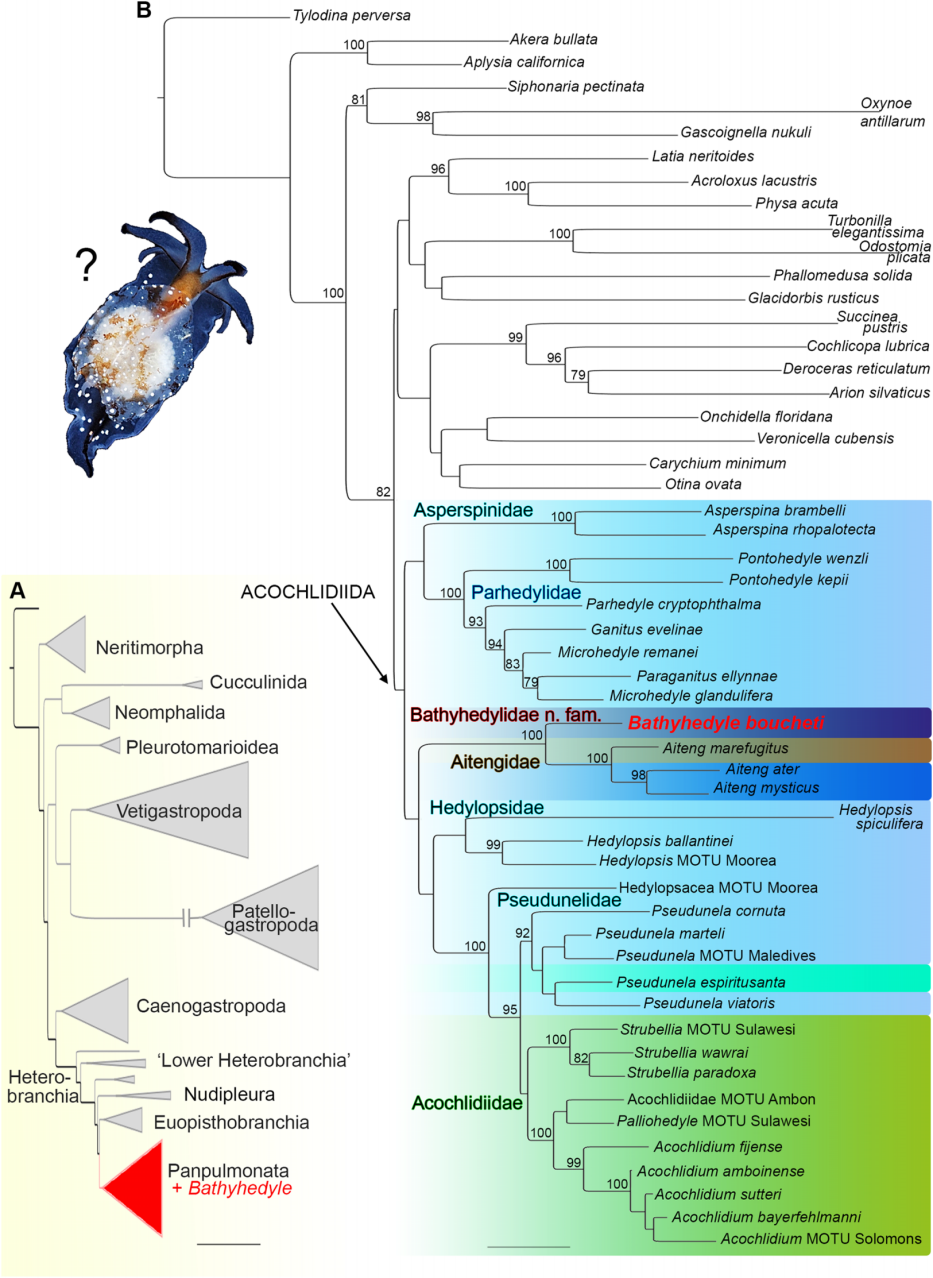
Phylogenetic hypothesis on the relationships of *Bathyhedyle* n. gen. (based on concatenated four marker dataset). (A) Initial RAxML-analyses including all major gastropod clades clustering *Bathyhedyle* n. gen. among Panpulmonata. (B) Relationships among panpulmonate Acochlidiida (RAxML, 1,000 bootstrap replicates). Color coding corresponds to habitats: brown, terrestrial; green, freshwater; turquoise, brackish; light blue, marine; blue, marine-amphibious; dark blue, deep sea.

In the initial phylogenetic analysis with expanded taxon sampling including all major gastropod clades (see Table S1), the unique deep-sea slug clustered within heterobranch panpulmonates (see Fig. 1A). In the refined analysis with targeted taxon sampling of Acochlidiida and their panpulmonate relatives (see Table S2), it was recovered as the sister group to amphibious and terrestrial Aitengidae with high bootstrap support (see Fig. 1B). Relationships among the major clades of Acochlidiida are otherwise poorly supported based on our data.

### Systematics

Subclass Heterobranchia
Subcohort Panpulmonata
Order Acochlidiida
SPF Acochlidioidea
Bathyhedylidae n. fam.

**Type genus:** *Bathyhedyle* n. gen.

**Diagnosis:** Macroscopic acochlidian deep-sea slug with foot at least three times broader than head, and longer than notum. Propodial tentacles present. Two pairs of cephalic tentacles of equal length. Small, black dorsolateral eyes, posterior to rhinophores.

*Bathyhedyle* n. gen.

**Type species:** *Bathyhedyle boucheti* n. sp., here designated.

**Diagnosis:** As for family.

**Etymology:** From Greek, *bathys*, meaning ‘deep,’ and *Hedyle*, the first genus of the Acochlidiida.

*Bathyhedyle boucheti* n. sp.

**Type material:** Holotype (MNHN IM-2000-27917), in 95% ethanol (lacking part of foot and pharynx), and mounted radula, deposited in the Muséum National d’Histoire Naturelle, Paris, France.

Paratype (ZSM Mol 20140455), histological section series, deposited in the Bavarian State Collection of Zoology, Munich, Germany.

**Type locality:** Off Mozambique, Mozambique Channel, transect off the mouth of the Zambeze, station CC3150, 19°31′S, 36°46′E, depth approx. 260 m.

**Diagnosis:** With the same characters of the family and additionally: width of head one-third of width of foot. Tips of cephalic tentacles black. Cephalic tentacles and tail with a dorsal black stripe. Head and dorsal aspect of cephalic tentacles brownish. Free, oval-shaped notum. Radular formula: 75 × 1.1.2.

**Etymology:** The species epithet honors the French malacologist Philippe Bouchet who co-organized the expedition that sampled the type material and who was central in recognizing the significance of the discovery.

### External morphology

**Figure 2:**
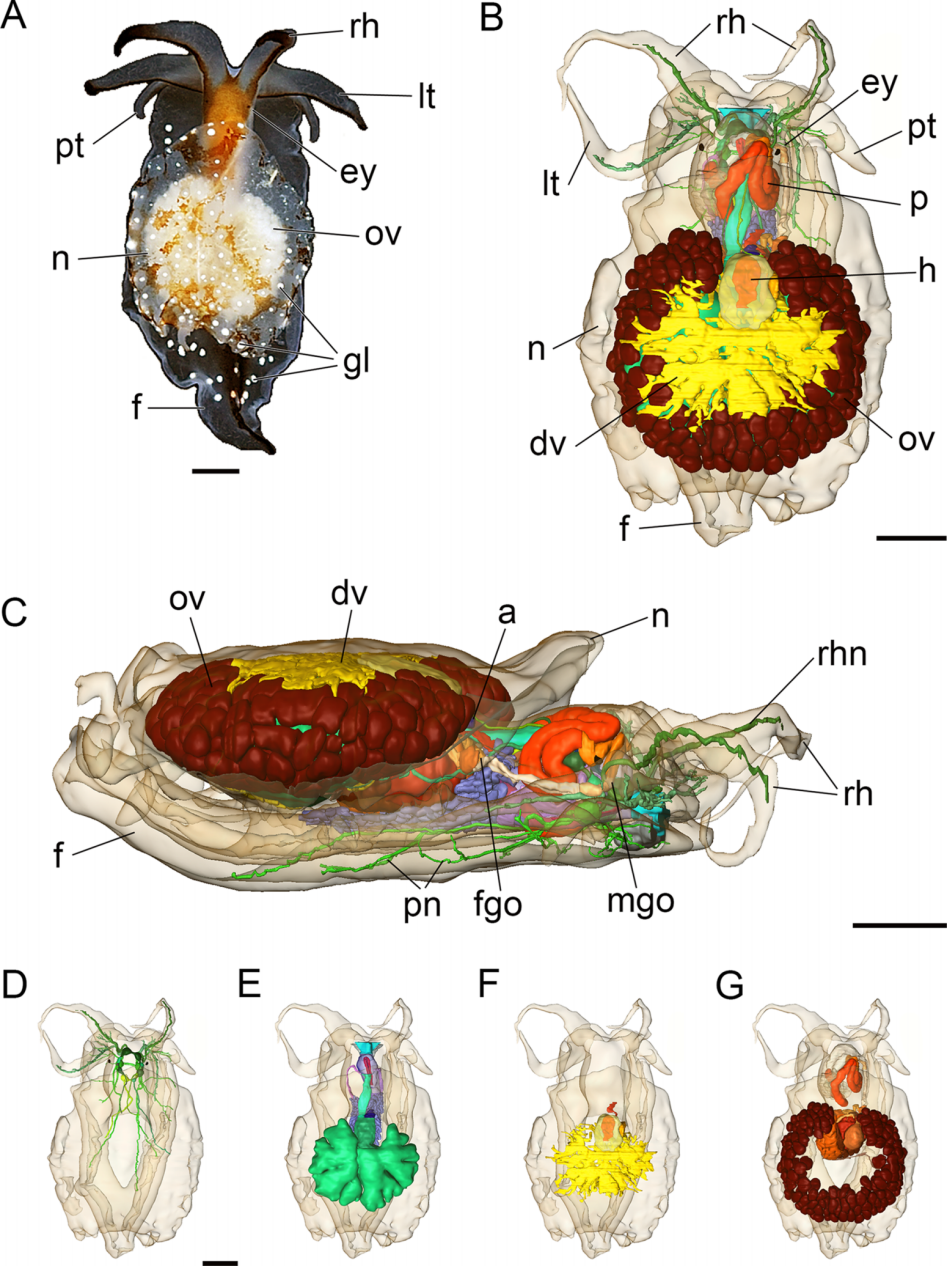
Photograph of a living specimen (A) and 3D reconstructions (B–G) of *Bathyhedyle boucheti* n. sp. (A) External morphology, dorsal view. (B) General microanatomy, dorsal view, (C) right view. (D–G) Positions of the organ systems, dorsal view; (D) central nervous system, (E) digestive system, (F) circulatory and excretory systems, (G) reproductive system. a, anus; dv, ‘dorsal vessel system’; ey, eye; f, foot; fgo, female gonopore; gl, subepidermal gland; h, heart; lt, labial tentacle; mgo, male gonopore; n, notum; ov, ovotestis; p, penis; pn, pedal nerve; pt, propodial tentacle; rh, rhinophore; rhn, rhinophoral nerve. Scale bars: (A–D) 1 mm, scale bar in (D) valid for (D–G). The interactive 3D model can be accessed by clicking on Fig. S1. Rotate model by dragging with left mouse button pressed, shift model: same action + ctrl (or change default action for left mouse button), zoom: use mouse wheel. Select or deselect (or change transparency of) components in the model tree, switch between prefab views or change surface visualization, (e.g. lightning, render mode, crop etc.). Interactive manipulation requires Adobe Reader 7 or higher.

**Figure 3:**
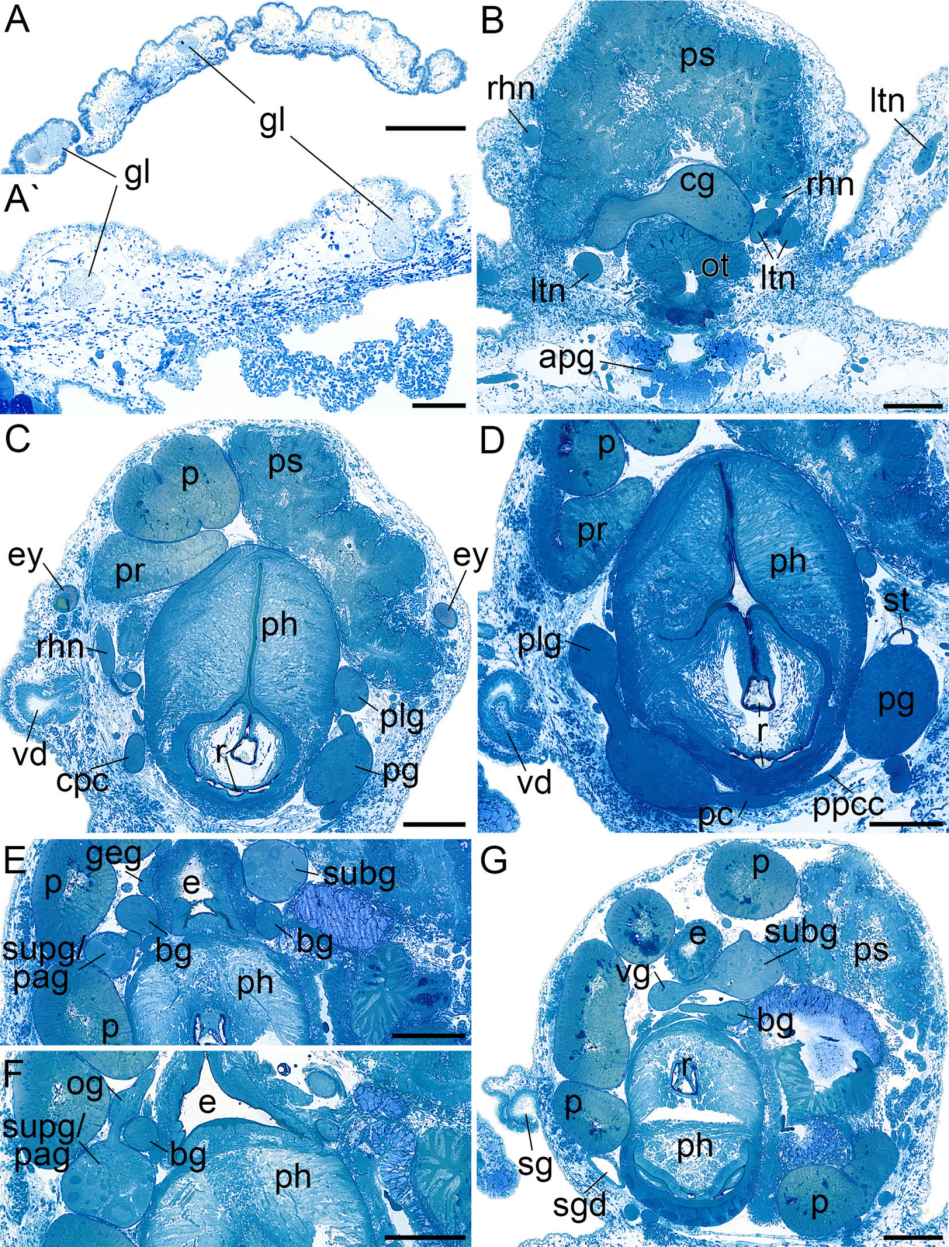
Histological cross sections of *B. boucheti* n. sp. (A, A′) Subepidermal glands. (B) Anterior pedal gland and cerebral ganglia. (C) Eyes and copulatory organs. (D) Parapedal commissure, statocyst and pharynx. (E) Subintestinal and fused supraintestinal/right parietal ganglia. (F) Osphradial ganglion. (G) Sperm groove and buccal ganglion. apg, anterior pedal gland; bg, buccal ganglion; cg, cerebral ganglion; cpc, cerebro-pedal connective; e, esophagus; ey, eye; geg, gastro-esophageal ganglion; gl, subepidermal gland; ltn, labial tentacle nerve; og, osphradial ganglion; ot, oral tube; p, penis; pc, pedal commissure; pg, pedal ganglion; ph, pharynx; plg, pleural ganglion; ppcc, parapedal commissure; pr, prostate; ps, penial sheath; r, radula; rhn, rhinophoral nerve; sg, external sperm groove; sgd, salivary gland duct; st, statocyst; subg, subintestinal ganglion; supg/pag, fused supraintestinal/right parietal ganglion; vd, internal vas deferens; vg, visceral ganglion. Scale bars: (A) 500 µm, (A′) 100 µm, (B–G) 200 µm.

The adult body length of living specimens is approx. 9 mm. The head bears two pairs of cephalic tentacles (Fig. 2A). The equally long labial tentacles and the rhinophores are cylindrical, solid and taper to the tip. Pigmented eyes are situated dorsolaterally just behind the rhinophores (Figs. 2A and 2B). The foot is three times the width of the head and tapers posteriorly. Propodial tentacles are present at the anterior of the foot. The head is brownish dorsally, the cephalic tentacles and the foot are semi-translucent. A black stripe extends along the dorsal aspect of the cephalic tentacles and dorsally along the posterior part of the foot (Fig. 2A). The free and oval-shaped notum is as wide as the foot or slightly broader and extends over two-thirds of the body length. Parts of the internal organs, such as the ovotestis and digestive gland are visible through the transparent notum. There are white glandular spots (Figs. 2A, 3A and 3A′) on the mantle and foot. A shell, gills and mantle cavity are absent. The head may be retracted partially (except the cephalic tentacles) under the notum when the animal is disturbed.

### Microanatomy

In addition to the figure plates, consider also the interactive 3D-model in Fig. S1.

#### Central nervous system

**Figure 4:**
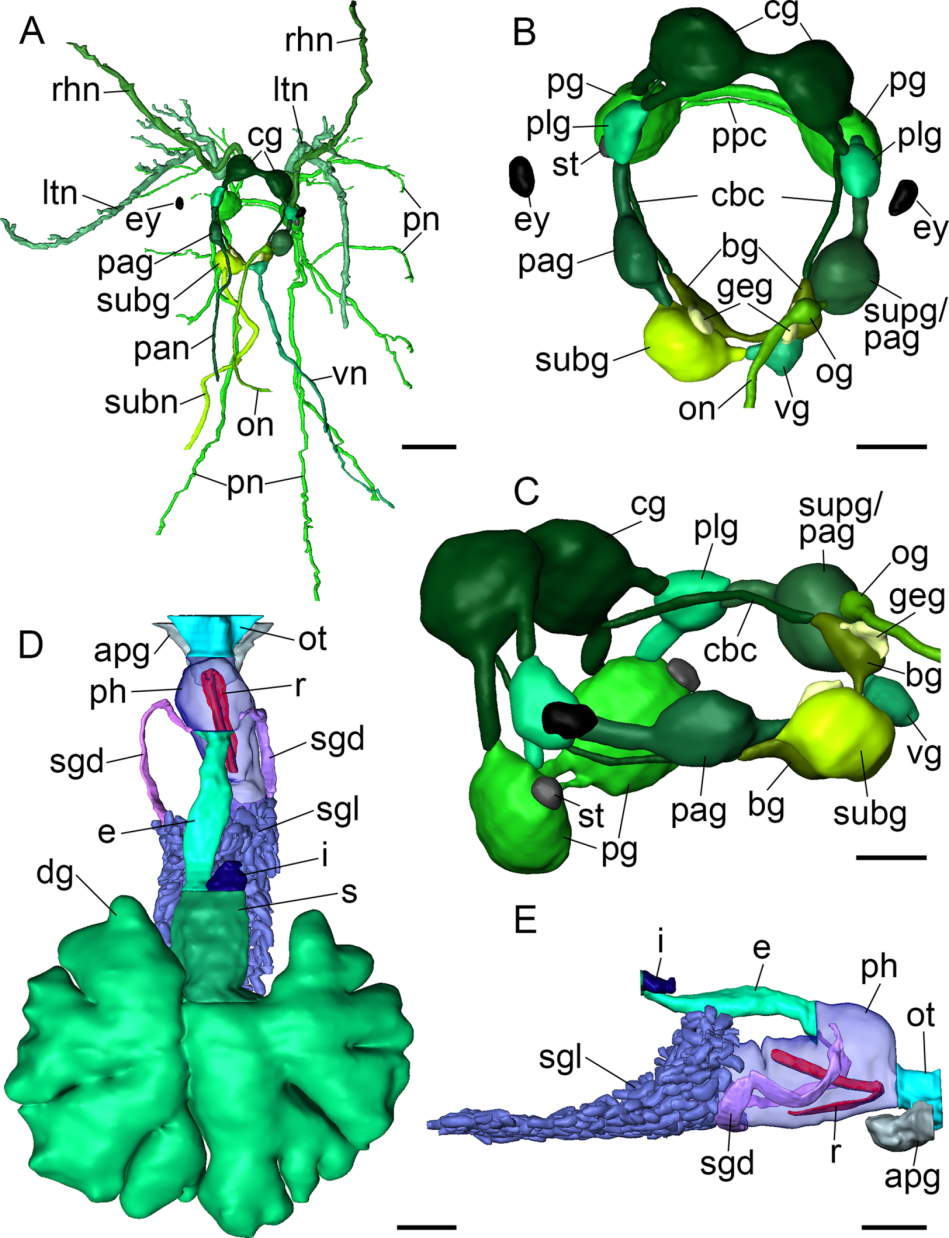
3D reconstructions of *B. boucheti* n. sp. (A) Central nervous system, dorsal view. (B, C) Central nervous system without nerves, dorsal and left views, respectively. (D) Digestive system, dorsal view. (E) Digestive system without digestive gland, right view. apg, anterior pedal gland; bg, buccal ganglion; cbc, cerebro-buccal connective; cg, cerebral ganglion; dg, digestive gland; e, esophagus; ey, eye; geg, gastro-esophageal ganglion; i, intestine; ltn, labial tentacle nerve; og, osphradial ganglion; on, osphradial nerve; ot, oral tube; pag, left parietal ganglion; pan, left parietal nerve; pg, pedal ganglion; ph, pharynx; plg, pleural ganglion; pn, pedal nerve; ppc, parapedal commissure; r, radula; rhn, rhinophoral nerve; s, stomach; sgd, salivary gland duct; sgl, salivary gland; st, statocyst; subg, subintestinal ganglion; subn, subintestinal nerve; supg/pag, fused supraintestinal/right parietal ganglion; vg, visceral ganglion; vn, visceral nerve. Scale bars: (A, D, E) 500 µm, (B) 200 µm, (C) 150 µm.

The central nervous system (Fig. 2D) is euthyneurous and pre-pharyngeal with paired cerebral, pedal, pleural, buccal and gastro-oesophageal ganglia, and with four distinct ganglia plus a presumed osphradial ganglion on the visceral loop (Fig. 4A). The cerebral, pedal and pleural ganglia form the pre-pharyngeal nerve ring. The thick labiotentacular and the rhinophoral nerves (Figs. 3B and 4A) emerge anteroventrally and anterodorsally, respectively, from each cerebral ganglion (approx. 260 μm) innervating the labial tentacles and the rhinophores. The eyes (Figs. 3C and 4B) (approx. 115 μm) are situated dorsolaterally at the level of the pleural ganglia. Precerebral accessory ganglia, rhinophoral and optic ganglia are lacking. The optic nerve was not detected. The pedal ganglia (approx. 280 μm) lie ventral to the cerebral ganglia (Fig. 4C). The pedal commissure is thinner and longer than the cerebral commissure and is flanked by a thin parapedal commissure (Figs. 3D and 4B). Three pedal nerves (Figs. 2C and 4A) emerge from each pedal ganglion (one anteroventrally and two posteriorly) innervating the foot. One statocyst with a statolith (Figs. 3D and 4C) is attached dorsally to each pedal ganglion. The pleural ganglia (approx. 150 μm) are connected to the ganglia of the visceral loop by short connectives (Figs. 3C, 3D, 4B and 4C). Four separate ganglia are situated on the visceral loop: the left parietal ganglion (Figs. 4A–4C) (approx. 235 μm), the subintestinal ganglion (Figs. 3E, 3G and 4A–4C) (approx. 250 μm), the small visceral ganglion (Figs. 3G, 4B and 4C) (approx. 120 μm) and the fused supraintestinal/right parietal ganglion (Figs. 3E, 3F, 4B and 4C) (approx. 250 μm). A small presumed osphradial ganglion (Figs. 3F, 4B and 4C) is attached to the fused supraintestinal/right parietal ganglion. The osphradial nerve innervates the right body wall under the notum, although no histologically differentiated osphradium could be detected. One nerve emerges from each ganglion on the visceral loop (except the fused supraintestinal/right parietal ganglion) extending to the posterior (Fig. 4A). Paired buccal ganglia (approx. 180 μm) are situated posterior to the pharynx and are linked by a short commissure ventral to the oesophagus (Figs. 3G and 4C). The cerebro-buccal connectives (Fig. 4C) emerge posteroventrally from the cerebral ganglia. A smaller gastro-oesophageal ganglion (Figs. 3E and 4C) (approx. 100 μm) is connected dorsally to each buccal ganglion.

#### Digestive system

**Figure 5:**
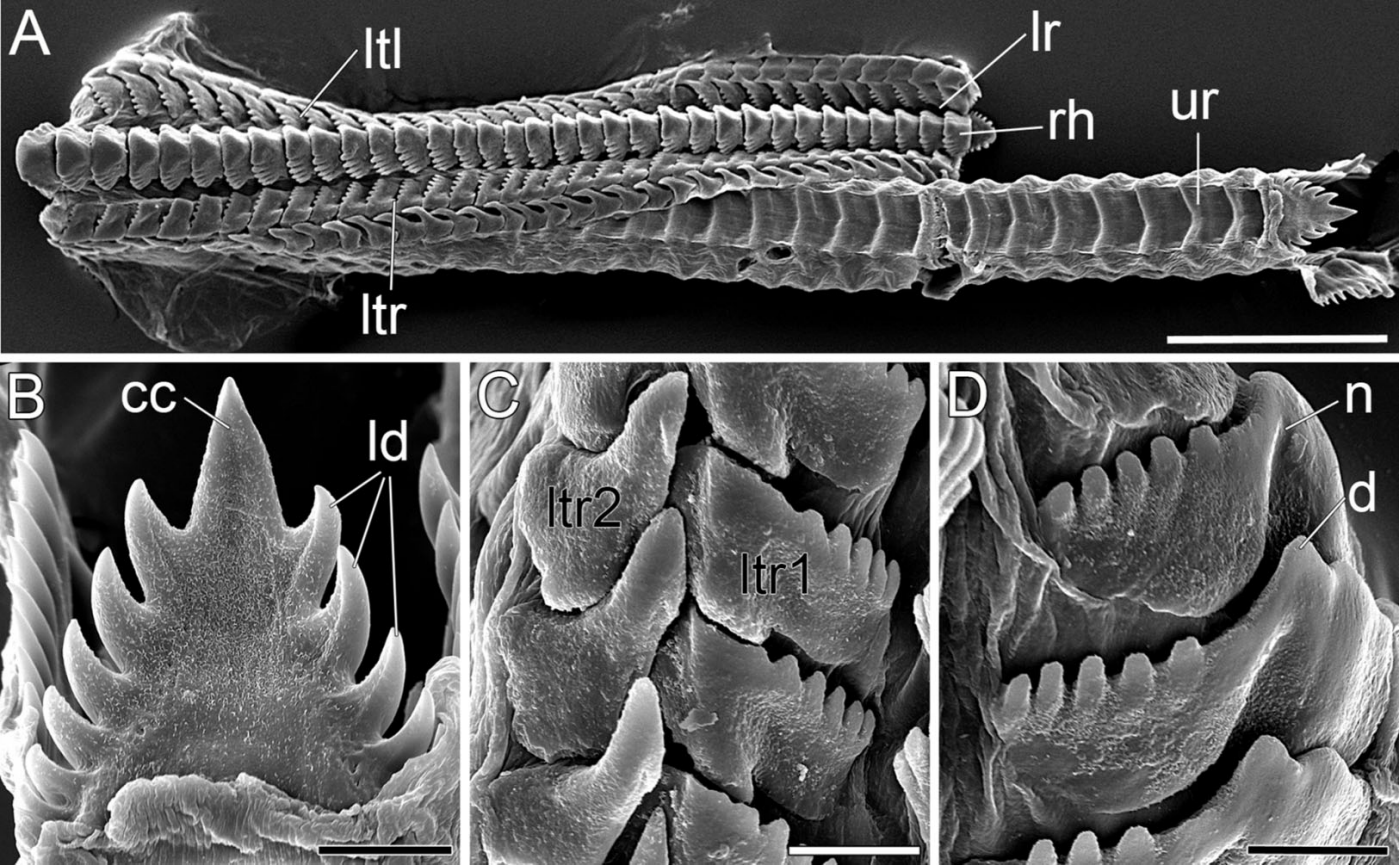
SEM micrographs of the radula of *B. boucheti* n. sp. (A) Upper and lower ramus. (B) Rachidian. (C) Right lateral teeth. (D) Left lateral teeth. cc, central cusp; d, denticle; ld, lateral denticle; lr, lower ramus; ltl, left lateral tooth; ltr1/2, right lateral teeth; n, notch; rh, rhachidian tooth; ur, upper ramus. Scale bars: (A) 100 µm, (B–D) 10 µm.

**Figure 6:**
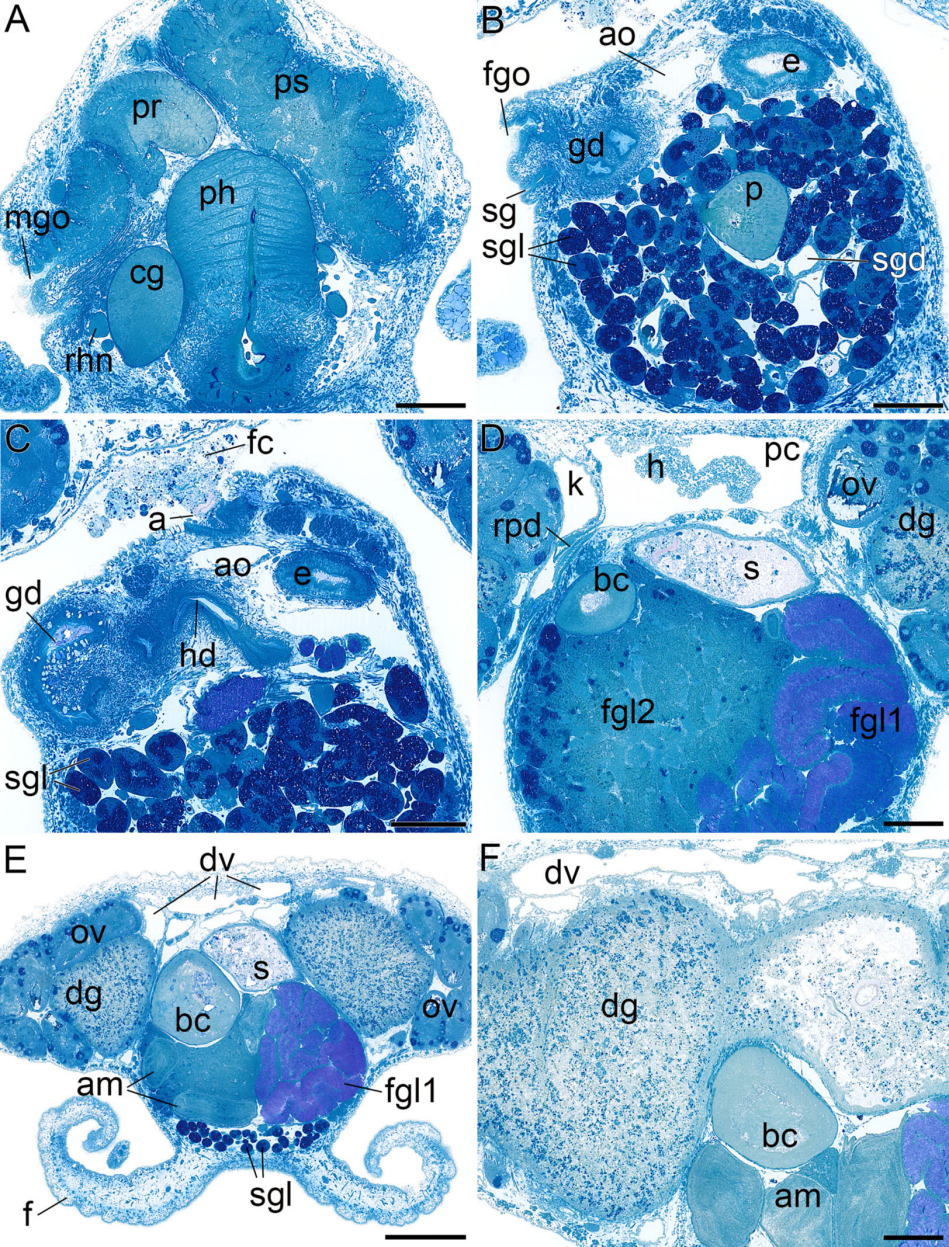
Histological cross sections of *B. boucheti* n. sp. (A) Male gonopore, prostate and penial sheath. (B) Female gonopore, aorta and salivary gland. (C) Anus and distal gonoduct. (D) Renopericardial duct, pericardium and heart. (E) ‘*Dorsal vessel system’ and female glands. (F) Digestive gland and ampulla. a, anus; am, ampulla; ao, aorta; bc, bursa copulatrix; cg, cerebral ganglion; dg, digestive gland; dv, ‘dorsal vessel system’; e, esophagus; f, foot; fc, feces; fgl1/2, female glands; fgo, female gonopore; gd, distal gonoduct; h, heart; k, kidney; mgo, male gonopore; ov, ovotestis; p, penis; pc, pericardium; ph, pharynx; pr, prostate; ps, penial sheath; rhn, rhinophoral nerve; rpd, renopericardial duct; s, stomach; sg, external sperm groove; sgl, salivary gland; sgd, salivary gland duct. Scale bars: (A–D, F) 200 µm, (E) 500 µm.

The mouth opens ventrally between the labial tentacles. The anterior pedal gland (Figs. 3B, 4D and 4E) discharges ventrally to the mouth. The oral tube (Figs. 3B, 4D and 4E) is short and unciliated. The muscular pharynx (Figs. 3C–3G, 4D and 4E) contains the radula (Figs. 3C and 3D). The radula is J-shaped (Fig. 4E) and approx. 1 mm long. The radular formula is 1.1.2 and comprises roughly 75 rows of teeth, approx. 35 of them on the lower ramus, 40 on the upper (Fig. 5A). Each row consists of a rachidian (Fig. 5B) and a single left lateral tooth (Fig. 5D), and two right lateral teeth (Fig. 5C). The rachidian is triangular with one large central cusp flanked by five smaller denticles on each side. The left lateral tooth is apparently formed by fusion of two teeth; is plate-like with one large denticle and up to seven smaller, pointed denticles along the anterior margin; a prominent notch along the posterior margin receives the large denticle of the following tooth (Fig. 5D). The inner right lateral tooth is plate-like with roughly six smaller denticles along the anterior margin. The outer right lateral tooth is narrow and bears a pointed denticle which corresponds to the large denticle of the left tooth. The oesophagus (Figs. 3E–3G, 4D and 4E) is long, ciliated and uncuticularized. One pair of large salivary glands consisting of numerous follicles (Figs. 4E, 6B, 6C and 6E) discharges via paired salivary gland ducts (Figs. 4D, 4E and 6B) into the posterior pharynx. Jaws and gizzard are lacking. No distinct stomach could be detected. The digestive gland extends almost to the posterior end of the mantle (Fig. 2E) and consists of several branches (Figs. 4D, 6E and 6F). The intestine is densely ciliated and short (Figs. 4D and 4E). The anus (Figs. 2C and 6C) opens dorsally on the right side of the body under the notum and slightly above and behind the female gonopore.

#### Circulatory and excretory systems

**Figure 7:**
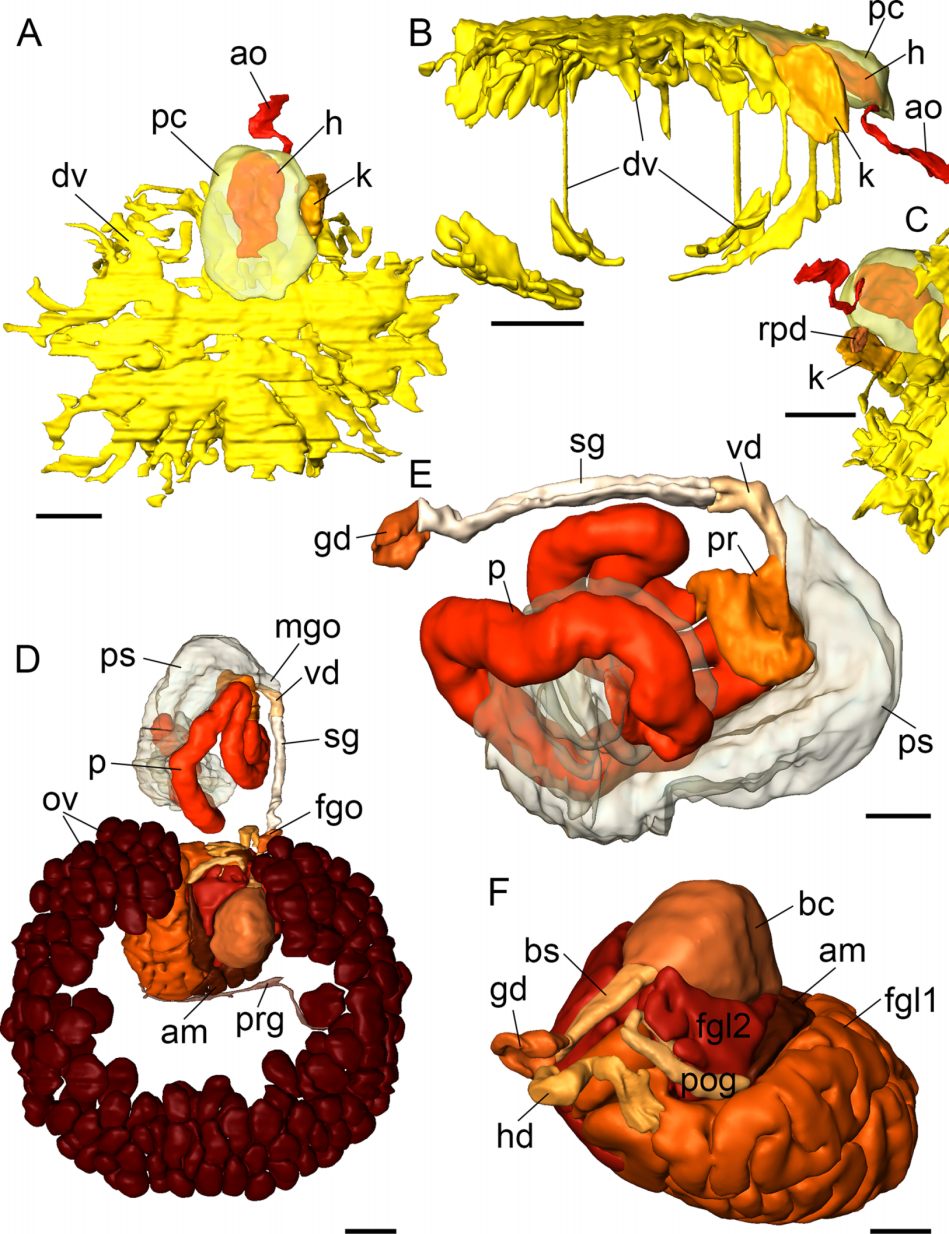
3D reconstructions of *B. boucheti* n. sp. (A–C) Circulatory and excretory systems. (A) Dorsal view, (B) right view, (C) renopericardial duct, ventral view. (D–F) Reproductive system. (D) Dorsal view, (E) copulatory organs, ventral view, (F) female glands and bursa copulatrix; left anterior view, compact female gland 1 is reconstructed as a solid mass rather than tubular. am, ampulla; ao, aorta; bc, bursa copulatrix; bs, bursa stalk; dv, ‘dorsal vessel system’; fgl1/2, female glands; fgo, female gonopore; gd, distal gonoduct; h, heart; hd, hermaphroditic duct; k, kidney; mgo, male gonopore; ov, ovotestis; p, penis; pr, prostate; ps, penial sheath; pc, pericardium; pog, postampullary gonoduct; prg, preampullary gonoduct; rpd, renopericardial duct; sg, external sperm groove; vd, internal vas deferens. Scale bars: (A–D) 500 µm, (E, F) 250 µm.

The circulatory and excretory systems (Fig. 2F) lie dorsal to the digestive and reproductive systems (Fig. 2C). The circulatory system consists of a broad, thin-walled pericardium surrounding a large heart (Figs. 6D and 7A–7C). The aorta (Figs. 6C and 7A–7C) emerges from the heart and extends anteriorly. A densely ciliated renopericardial duct (Figs. 6D and 7C) connects to the thin-walled kidney (Figs. 6D and 7A–7C). The kidney merges with an extensive system of ramified ‘dorsal vessels’ (Figs. 2B, 2C, 6E and 6F) lined with a very thin epithelium that extend mainly dorsal to the notum border, with a few extending also ventrally (Fig. 7B). The nephroduct and nephropore were not detected, but (considering the position of the kidney) the nephropore should be situated posterior to the intestine.

#### Reproductive system

The reproductive system (Fig. 2G) is hermaphroditic and monaulic. The ovotestis is situated dorsolaterally relative to the digestive gland (Figs. 2B and 2C) and consists of numerous follicles (Figs. 6D and 6E) forming an incomplete ring encircling the digestive gland and dorsal vessel system. Oocytes are arranged more in the outer part, spermatocytes mainly in the inner part of the follicles. Minute ducts drain the follicles and join to form the preampullary gonoduct (Fig. 7D). The ampulla (Figs. 6E, 6F and 7D) is large, tubular and filled with unorientated autosperm. Sperm heads are short. A receptaculum seminis is absent or not developed in the examined specimen. Two tubular female glands discharge into the postampullary gonoduct (Fig. 7F). The staining properties of the proximal female gland 1 is lilac and of the distal female gland 2 light blue (Figs. 6D and 6E). The latter is very compact and was reconstructed as a solid mass. The bursa copulatrix (Figs. 6D–6F and 7F) is large and sac-like and connected to the distal gonoduct by a long stalk. The distal gonoduct opens through the female gonopore (Figs. 2C, 6B and 7D) at the right side of the body slightly anterior to the anus. An external sperm groove (Figs. 3G, 6B, 7D and 7E) extends from the female gonopore to the right rhinophore and joins the short internal vas deferens (Figs. 3C, 3D, 7D and 7E). The latter connects to the small, tubular prostate (Figs. 3C, 3D, 6A and 7E). The long, muscular penis (Figs. 3C–3E, 7D and 7E) is thin, lacking a stylet or thorn, and partially surrounded by the penial sheath (Figs. 3B, 3C, 6A, 7D and 7E). The male gonopore (Figs. 2C, 6A and 7D) opens to the exterior at the base of the right rhinophore.

## Discussion

Surprisingly, the molecular results support the placement of the marine, deep-sea slug *B. boucheti* n. sp. in a clade within the usually coastal mesopsammic or limnic Acochlidiida. Even more fascinating is the sister group relationship with the amphibious and terrestrial Aitengidae.

Externally, the macroscopic *B. boucheti* n. sp. with two pairs of well-developed head tentacles and the oval-shaped notum differs greatly from typical, tiny and streamlined mesopsammic acochlidians (Schrödl & Neusser, 2010) or from recently discovered, compact and almost atentaculate Aitengidae (Neusser et al., 2011; Swennen & Buatip, 2009). However, the clade uniting Bathyhedylidae and Aitengidae is supported by several putative synapomorphies revealed by our micro-anatomical analyses, including the follicular salivary glands and ovotestis, and the well-developed, ramified ‘dorsal vessel system’ connected to the kidney (see Neusser et al., 2011). Features that serve to distinguish *Bathyhedyle* n. gen. from aitengids, and that justify its recognition at the rank of family include its drastically different ecology and a suite of external and internal characters that do not fit the current concept of Aitengidae, including the free, oval-shaped notum, the presence of well-developed cephalic and propodial tentacles and an external sperm groove, the absence of optic ganglia, and a radular formula of 1.1.2 (versus 1.1.1).

The morphological differences between *Bathyhedyle* n. gen. and *Aiteng* can be interpreted as adaptations to the very different ecologies. In the Aitengidae the cephalic tentacles are represented by a pair of more or less developed, rounded protuberances, and the visceral mass is fused with the foot along its entire length. The bulky body shape and subepidermal supporting cells provide stability to the body wall and are likely adaptations to its (semi)terrestrial lifestyle (Kano et al., 2015; Neusser et al., 2011). In contrast, the external morphology of *B. boucheti* n. sp. resembles that of large limnic distantly related Acochlidiidae (e.g. Brenzinger et al., 2011a; Haynes & Kenchington, 1991). Considering a marine mesopsammic acochlidian ancestor (Jörger et al., 2014a; Schrödl & Neusser, 2010), the external morphological and micro-anatomical features of *Bathyhedyle* n. gen. may be interpreted as independent adaptations to a benthic life style, including the large body size, broad foot, and two pairs of cephalic tentacles. Remarkably, apart from the limnic Acochlidiidae and (semi)terrestrial Aitengidae, all other acochlidians are microscopic and mesopsammic. *Bathyhedyle* n. gen. is the only known acochlidian with large, externally visible subepidermal glands. We interpret these as possible organs of defense. Also unique within the Acochlidiida are the long propodial tentacles, possibly reflecting the increased need for chemo- and mechanoreception, which support or replace the sensory role of eyes in a habitat characterized by decreasing light with increasing water depth (Warrant & Locket, 2004). Given the patchiness of species’ distributions (e.g. Gage & Tyler, 1991) and unpredictability of food resources in the deep sea, gigantism would enable animals to cover long distances during foraging and searching for mates (Clarke, 1960; Kaariainen & Bett, 2006). Thus, the comparatively large body size and benthic lifestyle of *B. boucheti* n. sp. may be interpreted as adaptations to the lonely, nutrient poor expanses of the deep sea. ‘Secondary gigantism’ in acochlidian species has been suggested to be a consequence of the habitat shift into brackish water, limnic and terrestrial environments (Jörger et al., 2014a). Introducing the deep water *Bathyhedylidae* n. fam., we present another, independent evolution of secondary gigantism within supposedly plesiomorphically minute acochlidians (Schrödl & Neusser, 2010).

*Bathyhedyle boucheti* n. sp. is the first documented marine benthic acochlidian species and represents a previously unknown habitat shift in acochlidian evolution from the marine mesopsammon back to a marine benthic lifestyle. Alternatively, the common ancestor of *Bathyhedylidae* n. fam. and Aitengidae may have been a macroscopic amphibious or even terrestrial species and the mesopsammic habitat was invaded secondarily. The presence of a largely ramified ‘dorsal vessel system’ in aitengids and *Bathyhedyle* n. gen. may support the hypothesis of a macroscopic ancestor. ‘Dorsal vessels’ in limnic *Acochlidium* represent an extended pericardium with podocytes, and thus can be interpreted as an enlarged site of ultrafiltration to produce large quantities of hypoosmotic urine; in contrast, the ‘dorsal vessels’ in the terrestrial *Aiteng marefugitus* represent an expanded kidney to enhance reabsorption of water and prevent desiccation (Timea P. Neusser, 2016, unpublished data). Neither desiccation nor osmotic stress poses a challenge to deep-sea molluscs. Thus, the ‘dorsal vessels’ in *Bathyhedyle* n. gen. may be a relic from an amphibious ancestor. Either way, the sister group relationship between a deep-sea species and a (semi)terrestrial lineage may be unique among invertebrates known so far and implies a remarkable level of evolutionary and ecological plasticity.

*Bathyhedyle boucheti* n. sp. is the first shell-less panpulmonate gastropod from the deep sea. This finding further contributes to the already remarkable habitat variation documented within Acochlidiida. The roughly 65 acochlidian species (Jörger et al., 2014a) succeeded in colonizing marine (shallow water to deep sea), brackish, limnic and terrestrial environments, whereas other panpulmonate taxa are exclusively marine (Pyramidelloidea), coastal or related to brackish waters (Sacoglossa), amphibious or terrestrial (Ellobioidea, Stylommatophora and Amphiboloidea), limnic (Hygrophila and Glacidorboidea) or inhabit marine shallow waters and comprise air-breathing species (Trimusculoidea, Otinoidea, Systellommatophora, Siphonarioidea). Acochlidian slugs thus encompass more fundamentally different habitats than any other ordinal-level taxon among the Panpulmonata, indeed among any other gastropod or mollusc taxon of comparable rank. The question then arises as to how the Acochlidiida could successfully invade a multitude of different habitats and demonstrate such a high degree of habitat flexibility.

The invasion of a new habitat requires not only adaptations to new physical conditions, but potential invaders must cope with new predators and competitors (e.g. Mordan & Wade, 2008). The morphological adaptations to a new habitat are quite well understood in Acochlidiida (Brenzinger et al., 2011a; Jörger et al., 2014a; Neusser, Jörger & Schrödl, 2011; Neusser & Schrödl, 2009); acochlidians show great flexibility in body shape and size, show extreme variation in reproductive morphology being the only euthyneuran gastropods with hermaphroditic and gonochoristic members, and have a remarkably adaptive excretory system (Neusser, Jörger & Schrödl, 2011; Schrödl & Neusser, 2010). Data on natural predators are limited to a few observations on *Pseudovermis* feeding on mesopsammic acochlids (Challis, 1969; T. Neusser, 2006, personal observation; Fize, 1961; Kowalevsky, 1901); additionally, defense mechanisms are unknown, apart from the mesopsammic or burrowing life mode of non-benthic species and the putative defensive glands in *Bathyhedyle* n. gen. Information about the food sources of Acochlidiida is patchy. Marine mesopsammic acochlidian species have never been observed during feeding. Jörger et al. (2014a) highlighted that the Acochlidiida comprise highly specialized feeders, e.g. *Strubellia* and *Acochlidium* feed on freshwater neritid egg capsules (Brenzinger et al., 2011b; Timea P. Neusser, 2016, unpublished data), *Aiteng ater* on insect pupae (Swennen & Buatip, 2009) and a yet undescribed *Aiteng* species from Papua New Guinea was observed to feed on molluscan egg masses of *Nerita trifasciata* Le Guillou, 1841 and *Siphonaria* sp. (Timea P. Neusser, 2016, unpublished data). The ancestor of at least aitengids and *Bathyhedylidae* n. fam. thus may have been oophagous, and we assume that *B. boucheti* n. sp. feeds on egg masses. The latter are available in all aquatic and terrestrial habitats in which molluscs occur. Therefore, we hypothesize that the specialization of feeding on eggs or other small and hulled protein-rich matter might explain the evolutionary flexibility in habitat choice of acochlidian species. Another gastropod group with highly diverse ecologies is the Neritopsina inhabiting marine shallow water (including submarine caves) and deep sea, brackish and freshwater systems, and terrestrial environments (Kano, Chiba & Kase, 2002; Ponder & Lindberg, 1997; Sasaki & Ishikawa, 2002). However, the reasons for their ability to successfully colonize this wide range of habitats are not known. A possible co-evolution of neritopsine and acochlidian species should be a focus of future research.

## Conclusions

Sister group relationships between deep-sea and shallow water species are known for several invertebrate taxa, among these, crustaceans (e.g. Hall & Thatje, 2009; Raupach et al., 2009; Tokuda et al., 2006) and molluscs (e.g. Eilertsen & Malaquias, 2015; Kano, Chiba & Kase, 2002; Kano et al., 2013; Oskars, Bouchet & Malaquias, 2015). However, the sister group relationship of the deep water *Bathyhedyle* n. gen. and the (semi)terrestrial Aitengidae is unusual within the Mollusca and to our knowledge unknown among other invertebrate taxa. We suspect the occurrence of small benthic acochlidian species in shallow waters; however, until now no such acochlidian species has yet been discovered. Our study highlights that the deep sea harbors significant but as yet unknown lineages awaiting discovery.

## Supplemental Information

10.7717/peerj.2738/supp-1 Supplemental Information 1 Interactive 3D model of *Bathyhedyle boucheti* n. sp. The interactive 3D model can be accessed by clicking on the figure. Rotate model by dragging with left mouse button pressed, shift model: same action + ctrl (or change default action for left mouse button), zoom: use mouse wheel. Select or deselect (or change transparency of) components in the model tree, switch between prefabricated views or change surface visualization (e.g. lightning, render mode, crop etc.). Interactive manipulation requires Adobe Acrobat Reader 7 or higher.

10.7717/peerj.2738/supp-2 Supplemental Information 2 Taxon sampling (species listed in alphabetic order with GenBank accession numbers) for initial phylogenetic placement of *Bathyhedyle* n. sp. among gastropods (dataset modified from Stöger et al., 2013). The protobranch bivalve Nucula sulcata was selected as outgroup.

10.7717/peerj.2738/supp-3 Supplemental Information 3 Taxon sampling (including GenBank Accession numbers) for phylogenetic relationships of *Bathyhedyle* n.sp. among panpulmonate gastropods with focus on Acochlidiida.

## Additional Information and Declarations

### DNA Deposition

The following information was supplied regarding the deposition of DNA sequences: GenBank Accession numbers: KX721048–KX721050.

### Data Deposition

The following information was supplied regarding data availability: Muséum National d’Histoire Naturelle, Paris, France (MNHN IM-2000-27917). Bavarian State Collection of Zoology, Munich, Germany (ZSM Mol 20140455).

### New Species Registration

The following information was supplied regarding the registration of a newly described species: Publication LSID: urn:lsid:zoobank.org:pub:F1DA5BD1-55D6-4F52-BEB5-446762706A52, Family name: urn:lsid:zoobank.org:act:4AC1FF05-EEEC-423F-A0A9-EB4DA636B219, Genus name: urn:lsid:zoobank.org:act:67E561CD-6B85-478B-98FB-BAAE49C10DAD, Species name: urn:lsid:zoobank.org:act:4B32A0B9-72BF-487C-8471-9C657D8E87A9.

## References

[ref-1] Altschul SF, Gish W, Miller W, Myers EW, Lipman DJ (1990). Basic local alignment search tool. Journal of Molecular Biology.

[ref-2] Ameziane N, Roux M (1997). Biodiversity and historical biogeography of stalked crinoids (Echinodermata) in the deep sea. Biodiversity & Conservation.

[ref-3] Benitez Villalobos F, Tyler PA, Young CM (2006). Temperature and pressure tolerance of embryos and larvae of the Atlantic seastars *Asterias rubens* and *Marthasterias glacialis* (Echinodermata: Asteroidea): potential for deep-sea invasion. Marine Ecology Progress Series.

[ref-4] Bouchet P, Duarte CM (2006). The magnitude of marine biodiversity. The Exploration of Marine Biodiversity–Scientific and Technological Challenges.

[ref-5] Bouchet P, Bary S, Héros V, Marani G, Héros V, Strong EE, Bouchet P (2016). How many species of molluscs are there in the world’s oceans, and who is going to describe them?. Tropical Deep-Sea Benthos.

[ref-6] Brenzinger B, Neusser TP, Glaubrecht M, Haszprunar G, Schrödl M (2011a). Redescription and three-dimensional reconstruction of the limnic acochlidian gastropod *Strubellia paradoxa* (Strubell, 1892) (Gastropoda: Euthyneura) from Ambon, Indonesia. Journal of Natural History.

[ref-7] Brenzinger B, Neusser TP, Jörger KM, Schrödl M (2011b). Integrating 3D microanatomy and molecules: natural history of the Pacific freshwater slug *Strubellia* Odhner, 1937 (Heterobranchia: Acochlidia), with description of a new species. Journal of Molluscan Studies.

[ref-8] Challis DA (1969). New species of *Pseudovermis* (Opisthobranchia: Aeolidacea) from New Zealand and the Solomon Islands. Transactions of the Royal Society New Zealand.

[ref-9] Clarke AH (1960). A giant ultraabyssal *Cocculina* (*C. superba*, n. sp.) from the Argentina Basin. Natural History Papers, National Museum of Canada.

[ref-10] Costello MJ, Coll M, Danovaro R, Halpin P, Ojaveer H, Miloslavich P (2010). A census of marine biodiversity knowledge, resources, and future challenges. PLoS ONE.

[ref-11] Edgar RC (2004). MUSCLE: multiple sequence alignment with high accuracy and high throughput. Nucleic Acids Research.

[ref-12] Eilertsen MH, Malaquias MAE (2015). Speciation in the dark: diversification and biogeography of the deep-sea gastropod genus *Scaphander* in the Atlantic Ocean. Journal of Biogeography.

[ref-13] Fize A (1961). Note préliminaire sur *Pseudovermis setensis* n. sp., mollusque opisthobranche eolidien mésopsammique de la côte languedocienne. Bulletin de la Société Zoologique de France.

[ref-14] Gage JD, Tyler PA (1991). Deep-Sea Biology: A Natural History of Organisms at the Deep-Sea Floor.

[ref-15] Grassle JM, Maciolek NJ (1992). Deep-sea species richness: regional and local diversity estimates from quantitative bottom samples. The American Naturalist.

[ref-16] Hall S, Thatje S (2009). Global bottlenecks in the distribution of marine Crustacea: temperature constraints in the family Lithodidae. Journal of Biogeography.

[ref-17] Haynes A, Kenchington W (1991). *Acochlidium fijiensis* sp. nov. (Gastropoda: Opisthobranchia: Acochlidiacea) from Fiji. Veliger.

[ref-18] Hessler RR, Thistle D (1975). On the place of origin of deep-sea lsopods. Marine Biology.

[ref-19] Jaume D, Duarte CM, Duarte CM (2006). General aspects concerning marine and terrestrial biodiversity. The Exploration of Marine Biodiversity–Scientific and Technological Challenges.

[ref-20] Jörger KM, Stöger I, Kano Y, Fukuda H, Knebelsberger T, Schrödl M (2010). On the origin of Acochlidia and other enigmatic euthyneuran gastropods, with implications for the systematics of Heterobranchia. BMC Evolutionary Biology.

[ref-21] Jörger KM, Brenzinger B, Neusser TP, Martynov AV, Wilson NG, Schrödl M (2014a). Panpulmonate habitat transitions: tracing the evolution of Acochlidia (Heterobranchia, Gastropoda). BioRxiv.

[ref-22] Jörger KM, Schrödl M, Schwabe E, Würzberg L (2014b). A glimpse into the deep of the Antarctic Polar Front–Diversity and abundance of abyssal molluscs. Deep Sea Research Part II: Topical Studies in Oceanography.

[ref-23] Kaariainen JI, Bett BJ (2006). Evidence for benthic body size miniaturization in the deep sea. Journal of the Marine Biological Association of the United Kingdom.

[ref-24] Kano Y, Chiba S, Kase T (2002). Major adaptive radiation in neritopsine gastropods estimated from 28S rRNA sequences and fossil records. Proceedings of the Royal Society B: Biological Sciences.

[ref-25] Kano Y, Fukumori H, Brenzinger B, Warén A (2013). Driftwood as a vector for the oceanic dispersal of estuarine gastropods (Neritidae) and an evolutionary pathway to the sunken-wood community. Journal of Molluscan Studies.

[ref-26] Kano Y, Neusser TP, Fukumori H, Jörger KM, Schrödl M (2015). Sea-slug invasion of the land. Biological Journal of the Linnean Society.

[ref-27] Karanovic I, Brandão SN (2015). Biogeography of deep-sea wood fall, cold seep and hydrothermal vent Ostracoda (Crustacea), with the description of a new family and a taxonomic key to living Cytheroidea. Deep Sea Research Part II: Topical Studies in Oceanography.

[ref-28] Klussmann-Kolb A, Dinapoli A, Kuhn K, Streit B, Albrecht C (2008). From sea to land and beyond-new insights into the evolution of euthyneuran Gastropoda (Mollusca). BMC Evolutionary Biology.

[ref-29] Kowalevsky A (1901). Les Hédylidés, étude anatomique. Memoires de l’Academie Imperiale des Sciences de St Petersbourg.

[ref-30] Lins LSF, Ho SYW, Wilson GDF, Lo N (2012). Evidence for Permo-Triassic colonization of the deep sea by isopods. Biology Letters.

[ref-31] Luft JH (1961). Improvements in epoxy resin embedding methods. Journal of Cell Biology.

[ref-32] Mestre NC, Thatje S, Tyler PA (2009). The ocean is not deep enough: pressure tolerances during early ontogeny of the blue mussel *Mytilus edulis*. Proceedings of the Royal Society B: Biological Sciences.

[ref-33] Miller MA, Pfeiffer W, Schwartz T (2010). Creating the CIPRES Science Gateway for inference of large phylogenetic trees.

[ref-34] Mordan PB, Wade CM, Ponder WF, Lindberg DR (2008). Heterobranchia II: the Pulmonata. Phylogeny and Evolution of the Mollusca.

[ref-35] Morse MP, Wilson WH, Stricker SA, Shinn GL (1994). Current knowledge of reproductive biology in two taxa of interstitial molluscs (class Gastropoda: order Acochlidiacea and class Aplacophora: order Neomeniomorpha). Reproduction and Development of Marine Invertebrates.

[ref-36] Neusser TP, Fukuda H, Jörger KM, Kano Y, Schrödl M (2011). Sacoglossa or Acochlidia? 3D reconstruction, molecular phylogeny and evolution of Aitengidae (Gastropoda: Heterobranchia). Journal of Molluscan Studies.

[ref-37] Neusser TP, Jörger KM, Schrödl M (2011). Cryptic species in tropic sandsz–interactive 3D anatomy, molecular phylogeny and evolution of meiofaunal Pseudunelidae (Gastropoda, Acochlidia). PLoS ONE.

[ref-38] Neusser TP, Schrödl M (2009). Between Vanuatu tides: 3D anatomical reconstruction of a new brackish water acochlidian gastropod from Espiritu Santo. Zoosystema.

[ref-39] Oskars TR, Bouchet P, Malaquias MAE (2015). A new phylogeny of the Cephalaspidea (Gastropoda: Heterobranchia) based on expanded taxon sampling and gene markers. Molecular Phylogenetics and Evolution.

[ref-40] Ponder WF, Lindberg DR (1997). Towards a phylogeny of gastropod molluscs: analysis using morphological characters. Zoological Journal of the Linnean Society.

[ref-41] Poore GCB, Avery L, Błażewicz-Paszkowycz M, Browne J, Bruce NL, Gerken S, Glasby C, Greaves E, McCallum AW, Staples D, Syme A, Taylor J, Walker-Smith G, Warne M, Watson C, Williams A, Wilson RS, Woolley S (2015). Invertebrate diversity of the unexplored marine western margin of Australia: taxonomy and implications for global biodiversity. Marine Biodiversity.

[ref-42] Raupach MJ, Mayer C, Malyutina M, Wägele J-W (2009). Multiple origins of deep-sea Asellota (Crustacea: Isopoda) from shallow waters revealed by molecular data. Proceedings of the Royal Society of London B: Biological Sciences.

[ref-43] Rex MA, McClain CR, Johnson NA, Etter RJ, Allen JA, Bouchet P, Warén A (2005). A source-sink hypothesis for abyssal biodiversity. The American Naturalist.

[ref-44] Richardson KC, Jarett L, Finke EH (1960). Embedding in epoxy resins for ultrathin sectioning in electron microscopy. Stain Technology.

[ref-45] Ruthensteiner B (2008). Soft part 3D visualization by serial sectioning and computer reconstruction. Zoosymposia.

[ref-46] Ruthensteiner B, Heß M (2008). Embedding 3D models of biological specimens in PDF publications. Microscopy Research and Technique.

[ref-47] Sasaki T, Ishikawa H (2002). The first occurrence of a neritopsine gastropod from a phreatic community. Journal of Molluscan Studies.

[ref-48] Schrödl M (2003). Sea Slugs of Southern South America.

[ref-49] Schrödl M (2014). Time to say “Bye-bye Pulmonata?”. Spixiana.

[ref-50] Schrödl M, Bohn JM, Brenke N, Rolán E, Schwabe E (2011a). Abundance, diversity, and latitudinal gradients of southeastern Atlantic and Antarctic abyssal gastropods. Deep Sea Research Part II: Topical Studies in Oceanography.

[ref-51] Schrödl M, Jörger KM, Klussmann-Kolb A, Wilson NG (2011b). Bye bye “Opisthobranchia”! A review on the contribution of mesopsammic sea slugs to euthyneuran systematics. Thalassas.

[ref-52] Schrödl M, Neusser TP (2010). Towards a phylogeny and evolution of Acochlidia (Mollusca: Gastropoda: Opisthobranchia). Zoological Journal of the Linnean Society.

[ref-53] Schüller M, Brandt A, Ebbe B (2013). Editorial: Diversity of Southern Ocean deep-sea benthos between cosmopolitism and cryptic speciation: new species from the ANDEEP expeditions. Zootaxa.

[ref-54] Schwabe E, Bohn JM, Engl W, Linse K, Schrödl M (2007). Rich and rare—first insights into species diversity and abundance of Antarctic abyssal Gastropoda (Mollusca). Deep Sea Research Part II: Topical Studies in Oceanography.

[ref-55] Smith AB, Stockley B (2005). The geological history of deep-sea colonization by echinoids: roles of surface productivity and deep-water ventilation. Proceedings of the Royal Society B: Biological Sciences.

[ref-56] Smith KE, Thatje S (2012). The secret to successful deep-sea invasion: does low temperature hold the key?. PLoS ONE.

[ref-57] Stamatakis A (2014). RAxML version 8: a tool for phylogenetic analysis and post-analysis of large phylogenies. Bioinformatics.

[ref-58] Stöger I, Sigwart JD, Kano Y, Knebelsberger T, Marshall BA, Schwabe E, Schrödl M (2013). The continuing debate on deep molluscan phylogeny: evidence for Serialia (Mollusca, Monoplacophora plus Polyplacophora). BioMed Research International.

[ref-59] Swedmark B (1968). Deux espèces nouvelles d’acochlidiacées (mollusques opisthobranches) de la faune interstitielle marine. Cahiers de Biologie Marine.

[ref-60] Swennen CK, Buatip S (2009). *Aiteng ater*, new genus, new species, an amphibious and insectivorous sea slug that is difficult to classify (Mollusca: Gastropoda: Opisthobranchia: Sacoglossa(?): Aitengidae, new family). The Raffles Bulletin of Zoology.

[ref-61] Talavera G, Castresana J (2007). Improvement of phylogenies after removing divergent and ambiguously aligned blocks from protein sequence alignments. Systematic Biology.

[ref-62] Thubaut J, Puillandre N, Faure B, Cruaud C, Samadi S (2013). The contrasted evolutionary fates of deep-sea chemosynthetic mussels (Bivalvia, Bathymodiolinae). Ecology and Evolution.

[ref-63] Tokuda G, Yamada A, Nakano K, Arita N, Yamasaki H (2006). Occurrence and recent long-distance dispersal of deep-sea hydrothermal vent shrimps. Biology Letters.

[ref-64] Tyler PA, Young CM, Clarke A (2000). Temperature and pressure tolerances of embryos and larvae of the Antarctic sea urchin *Sterechinus neumayeri* (Echinodermata: Echinoidea): potential for deep-sea invasion from high latitudes. Marine Ecology Progress Series.

[ref-65] Wägele H, Klussmann-Kolb A, Vonnemann V, Medina M, Ponder WF, Lindberg D (2008). Heterobranchia I: the Opisthobranchia. Phylogeny and Evolution of the Mollusca.

[ref-66] Warrant EJ, Locket NA (2004). Vision in the deep sea. Biological Reviews.

