# Supplementary material for: The unique deep sea—land connection: interactive 3D visualization and molecular phylogeny of Bathyhedyle boucheti n. sp. (Bathyhedylidae n. fam.)—the first panpulmonate slug from bathyal zones
Source: PeerJ. 2016 Dec 6;4:e2738. doi: 10.7717/peerj.2738 (PMC5144724; doi:10.7717/peerj.2738)
Supplement: Supplemental Information 1 — The interactive 3D model can be accessed by clicking on the figure. Rotate model by dragging with left mouse button pressed, shift model: same action + ctrl (or change default action for left mouse button), zoom: use mouse wheel. Select or deselect (or change transparency of) components in the model tree, switch between prefabricated views or change surface visualization (e.g. lightning, render mode, crop etc.). Interactive manipulation requires Adobe Acrobat Reader 7 or higher. [file peerj-04-2738-s001.pdf]

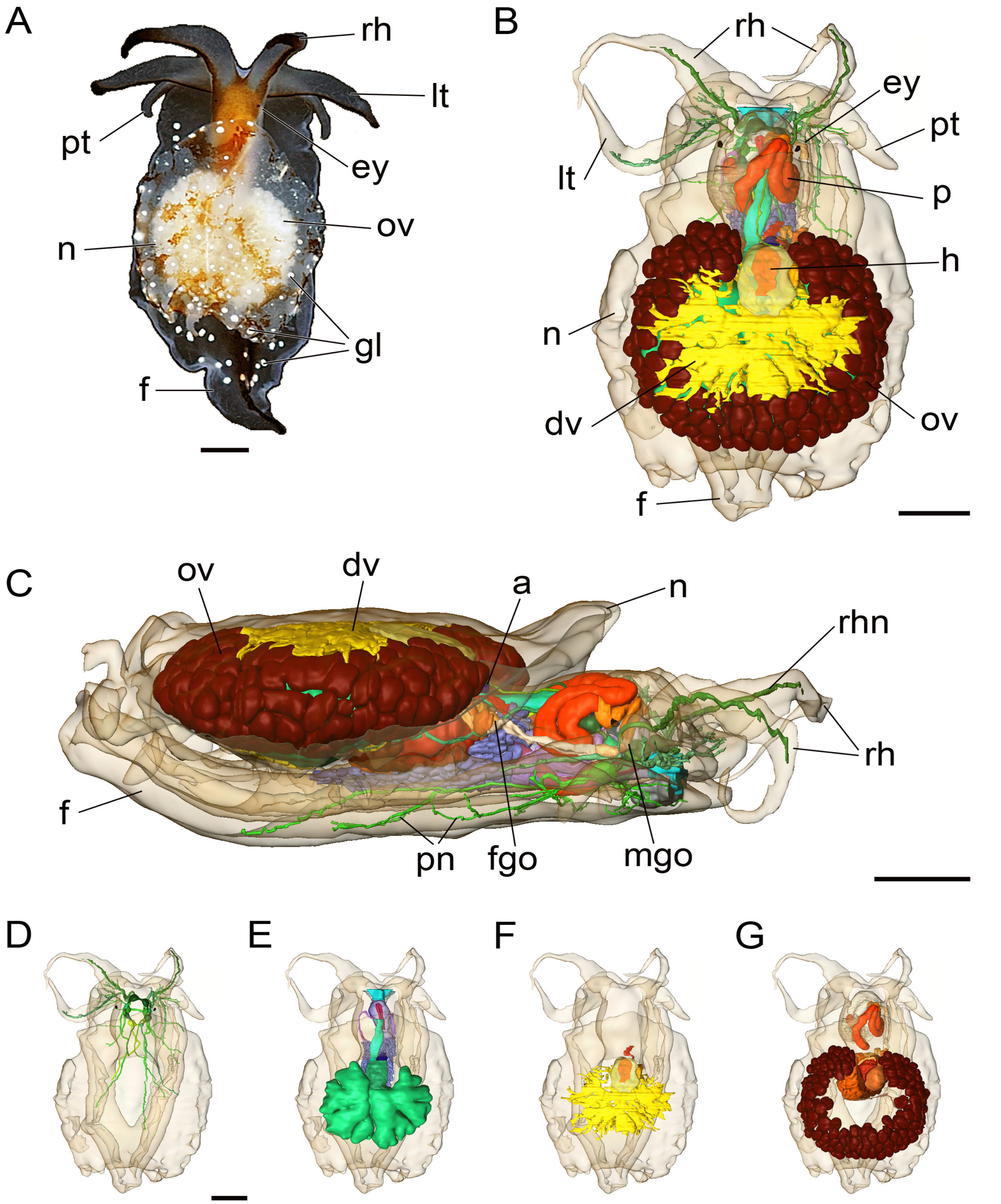

Supplementary Figure S1: Photograph of a living specimen (A) and 3D reconstructions (B-G) of *Bathyhedyle boucheti* n. sp. (**A**) External morphology, dorsal view. (**B**) General microanatomy, dorsal view, (**C**) right view. (**D-G**) Positions of the organ systems, dorsal view; (**D**) central nervous system, (**E**) digestive system, (**F**) circulatory and excretory systems, (**G**) reproductive system. a, anus; dv, 'dorsal vessel system'; ey, eye; f, foot; fgo, female gonopore; gl, subepidermal gland; h, heart; lt, labial tentacle; mgo, male gonopore; n, notum; ov, ovotestis; p, penis; pn, pedal nerve; pt, propodial tentacle; rh, rhinophore; rhn, rhinophoral nerve. Scale bars: (**A-D**) 1 mm, scale bar in (**D**) valid for (**D-G**).

The interactive 3D model can be accessed by clicking on Supplementary Figure S1. Rotate model by dragging with left mouse button pressed, shift model: same action + ctrl (or change default action for left mouse button), zoom: use mouse wheel. Select or deselect (or change transparency of) components in the model tree, switch between prefab views or change surface visualization, (e.g. lightning, render mode, crop etc.). Interactive manipulation requires Adobe Reader 7 or higher.
