# Supplementary material for: The unique deep sea—land connection: interactive 3D visualization and molecular phylogeny of Bathyhedyle boucheti n. sp. (Bathyhedylidae n. fam.)—the first panpulmonate slug from bathyal zones
Source: PeerJ. 2016 Dec 6;4:e2738. doi: 10.7717/peerj.2738 (PMC5144724; doi:10.7717/peerj.2738)
Supplement: Supplemental Information 2 — The protobranch bivalve Nucula sulcata was selected as outgroup. [file peerj-04-2738-s002.pdf]

Table S1:

	Species	18S	28S	16S	COI
Bivalvia	*Nucula sulcata*	DQ279937	DQ279960	DQ280029	DQ280017
Gastropoda	*Acmaea mitra*	AB282760	AB282781	AB106518	AB238459
	Addisonia lateralis	KF527260	KF527269	no data	no data
	Alcadia dysonia	DQ093428	DQ279974	DQ093469	no data
	Anatoma euglypta	AY923897	no data	no data	AY923934
	Aperostoma palmeri	DQ093435	DQ279983	DQ093479	DQ093523
	Aplysia californica	AY039804	AY026366	AF192295	AF077759
	Arion silvaticus	AY145365	AY145392	AY947380	AY987918
	Balcis eburnea	AF120519	AF120576	DQ280051	AF120636
	Bathyacmaea nipponica	AB282772	AB282793	AB238451	AB238588
	Bathydoris clavigera	AY165754	AY427444	AF249222.1	AF249808.1
	Bathymargarites symplector	DQ093433	DQ279982	DQ093477	DQ093521
	Bayerotrochus midas	AF120510	DQ093453	DQ093474	AY296820
	Bolinus brandaris	DQ279944	DQ279986	DQ280052	DQ280020
	Cavolinia uncinata	DQ237964	DQ237983	no data	DQ237997
	Cellana nigrolineata	DQ013353	DQ279971	DQ093467	DQ093515
	Chilina sp.	EF489338.1	EF489357.1	EF489305.1	EF489382.1
	Cima sp.	FJ917206.1	FJ917228.1	FJ917260.1	no data
	Coccopigya punctoradiata	AB282774	AB282795	AB238453	AB238590
	Cocculina messingi	AF120508	AY377696	AY377624	AY377731
	Crepidula fornicata	AY377660	JF750968	AY377625	AF353154
	Cyathermia naticoides	DQ093430	DQ279977	DQ093472	DQ093518
	Depressigyra globulus	DQ093431	DQ279978	DQ093473	DQ093519
	Diodora graeca	AF120513	DQ279980	DQ093476	AF120632
	Entemnotrochus adansonianus	AF120509	DQ279979	AY377621	L78910
	Eoacmaea conoidalis	AB282757	AB282778	AB238375	AB238505
	Erginus sybaritica	AB282761	AB282782	AB238350	AB238461
	Eulepetopsis vitrea	DQ093427	DQ279972	DQ093468	DQ093516
	Gascoignella nukuli	HQ168427	HQ168440	HQ168414	HQ168452
	Glacidorbis rusticus	FJ917211	FJ917227	FJ917264	FJ917284
	Haliotis tuberculata	AF120511	GQ160635	AY377622	AY377729
	Hedylopsis ballantinei	HQ168429	HQ168442	HQ168416	HQ168454
	Lepeta caeca	AB282759	AB282780	AB238347	AB238458
	Lepetodrilus elevatus	DQ093432	AY145413	DQ093475	DQ093520
	Leptogyropsis inflata	AB365313	no data	no data	AB365258
	Littorina littorea	DQ093437	DQ279985	DQ093481	DQ093525
	Lottia gigantea	AB282762	AB282783	AB106498	AB238466
	Micromelo undatus	DQ093443	DQ927214	DQ093487	DQ974653
	Mikadotrochus beyrichii	AM048636	AM048695	no data	AM049331
	Nacella magellanica	AB282769	AB282790	AB238433	EU870985

	Neomphalus fretterae	AY090806	no data	no data	no data
	Nerita funiculata	DQ093429	DQ279976	DQ093471	DQ093517
	Neritilia rubida	no data	AB087190	no data	AB102712
	Neritopsis radula	no data	AB087186	no data	no data
	Nipponacmea concinna	DQ013354	no data	AB106511	AB238486
	Notocrater houbricki	L78881	no data	no data	AY296822
	Onchidella sp.	DQ093441	DQ279992	DQ093485	DQ093529
	Ophicardelus ornatus	DQ093442	DQ256740	DQ093486	DQ093530
	Orbitestella vera	FJ917207	FJ917239	FJ917250	FJ917268
	Otina ovata	EF489344	EF489363	EF489310	EF489389
	Paralepetopsis sp.	FJ977635	FJ977665	FJ977699	FJ977752
	Patella vulgata	AB282770	AB282791	AB238445	AB238580
	Pectinodonta rhyssa	AB282773	AB282794	AB238452	AB238589
	Peltospira delicata	AY923893	FJ977684	FJ977716	AY923931
	Phenacolepas osculans	AY923890	no data	no data	AY923928
	Philine aperta	DQ093438	DQ279988	DQ093482	JN825187
	Pisulina adamsiana	no data	AB087191	no data	no data
	Pleurobranchus peroni	AY427494	AY427455	EF489331.1	DQ237993.1
	Pomacea bridgesi	AF055646	DQ279984	DQ093480	DQ093524
	Puncturella conica	KF527257	KF527266	KF527251	no data
	Pyropelta sp.	FJ977636	FJ977666	FJ977700	FJ977753
	Raphitoma linearis	DQ279945	DQ279987	DQ280053	KR084567
	Rictaxis punctocaelatus	EF489346	EF489370.1	GQ845193.1	EF489393.1
	Runcina africana	DQ923466	DQ927240.1	KJ022780	DQ974680.1
	Phallomedusa solida	DQ093440	DQ279991	DQ093484	DQ093528
	Scissurella coronata	AM048637	AM048696	no data	no data
	Scutellastra optima	AB282771	AB282792	AB106482	AB238585
	Seguenzia antarctica	KF527261	KF527270	KF527253	no data
	Sinezona confusa	AF120512	DQ279981	no data	no data
	Siphonaria pectinata	X91973	DQ279993	AY377627	AF120638
	Theodoxus fluviatilis	AF120515	DQ279975	DQ093470	AF120633
	Titiscania limacina	KF527262	KF527271	KF527254	no data
	Tricolia variabilis	AB365304	no data	no data	AB365219
	Truncatella guerini	AF120518	AF120575	EU233814	AF120635
	Turbonilla elegantissima	GU331941	GU331931	GU331951	GU331960
	Tylodina perversa	AY427496	AY427458	FJ917424	AF249809
	Valvata piscinalis	FJ917222	FJ917224	FJ917248	FJ917267
	Viviparus georginaus	AF120516	AF120574	AY377626	AF120634
