# Supplementary material for: The unique deep sea—land connection: interactive 3D visualization and molecular phylogeny of Bathyhedyle boucheti n. sp. (Bathyhedylidae n. fam.)—the first panpulmonate slug from bathyal zones
Source: PeerJ. 2016 Dec 6;4:e2738. doi: 10.7717/peerj.2738 (PMC5144724; doi:10.7717/peerj.2738)
Supplement: Supplemental Information 3 [file peerj-04-2738-s003.pdf]

Table S2:

Higher taxon	Species	18S rRNA	28S rRNA	16S rRNA	COI
**EUOPISTHOBRANCHIA**					
	Tylodina perversa	AY427496	AY427458	FJ917424	AF249809
	Akera bullata	AY427502	AY427466	AF156127	AF156143
	Aplysia californica	AY039804	AY026366	AF192295	AF077759
**PANPULMONATA**					
Sacoglossa					
Oxynoacea	*Oxynoe antillarum*	FJ917441	FJ917466	FJ917425	FJ917483
Plakobranchacea	*Gascoignella nukuli*	HQ168427	HQ168440	HQ168414	HQ168452
Siphonarioidea					
Siphonariidae	*Siphonaria pectinata*	HQ659934	DQ279993	AY377627	HQ660000
Pyramidelloidea					
Odostomiinae	*Odostomia plicata*	GU331938	GU331928	GU331948	GU331957
Turbonillinae	*Turbonilla elegantissima*	GU331941	GU331931	GU331951	GU331960
Glacidorboidea					
Glacidorbidae	*Glacidorbis rusticus*	FJ917211	FJ917227	FJ917264	FJ917284
Amphiboloidea					
Phallomedusidae	*Phallomedusa solida*	DQ093440	DQ279991	DQ093484	DQ093528
Hygrophila					
Latiidae	*Latia neritoides*	EF489339	EF489359	EF489307	EF489384
Acroloxidae	*Acroloxus lacustris*	AY282592	EF489364	EF489311	AY282581
Physidae	*Physa acuta*	AY282600	EF489368	AY651219	AY282589
**EUPULMONATA**					
Systellommatophora					
Odostomiinae	*Veronicella cubensis*	HQ659991	DQ897670	HQ659925	HQ660057
Onchidiidae	*Onchidella floridana*	AY427521	AY427486	EF489317	EF489392
Ellobioidea					
Otinidae	*Otina ovata*	EF489344	EF489363	EF489310	EF489389
Ellobiidae	*Carychium minimum*	EF489341	EF489361	EF489308	EF489386
Stylommatophora					
Arionoidea	*Arion silvaticus*	AY145365	AY145392	DQ465822	AF513018
Limacoidea	*Deroceras reticulatum*	AY145373	AY145404	AF238045	AF239734
Elasmognatha	*Succinea putris*	HQ659993	AY014057	HQ659927	HQ660059
Orthurethra	*Cochlicopa lubrica*	GU331944	GU331934	GU331954	GU331963
**Acochlidiida**					
Hedylopsidae	*Hedylopsis spiculifera*	HQ168430	HQ168443	HQ168417	HQ168455
	Hedylopsis ballantinei	HQ168429	HQ168442	HQ168416	HQ168454
	Hedylopsis MOTU Moorea	KF709276	no data	KF709247	KF709354
Hedylopsacea indet.	Hedylopsacea MOTU Moorea	KF709277	KF709320	no data	KF709355
Pseudunelidae	*Pseudunela cornuta*	JF819754	KF709321	JF819748	JF819774
	Pseudunela viatoris	JF819751	KF709322	JF819741	JF819766
	Pseudunela marteli	HQ168431	HQ168444	HQ168418	HQ168456
	Pseudunela MOTU Maledives	KF709279	KF709324	KF709249	KF709357
	Pseudunela espiritusanta	JF819755	KF709325	JF819749	JF819775
Acochlidiidae	*Strubellia paradoxa*	HQ168432	HQ168445	HQ168419	HQ168457
	Strubellia wawrai	KF709281	KF709327	JF819736	JF819764
	Strubellia MOTU Sulawesi	KF709282	KF709328	JF819740	JF819765
	Acochlidiidae MOTU Ambon	KF709283	KF709329	KF709250	KF709358
	Palliohedyle MOTU Sulawesi	KF709284	JF828039	JF828040	JF828032
	Acochlidium fijense	HQ168433	HQ168446	HQ168420	HQ168458
	Acochlidium amboinense	KF709285	KF709330	KF709251	KF709359
	Acochlidium bayerfehlmanni	KF709286	no data	KF709252	KF709360
	Acochlidium sutteri	KF709287	KF709331	KF709253	KF709361

	Acochlidium MOTU Solomons	KF709290	KF709334	KF709257	KF709365
Aitengidae	*Aiteng ater*	JF828036	JF828037	JF828038	JF828031
	Aiteng mysticus	HQ168428	HQ168441	HQ168415	HQ168453
	Aiteng marefugitus	AB914671	AB914672	AB914673	AB914674
Bathyhedylidae	*Bathyhedyle boucheti*	KX721049	KX721050	KX721048	no data
Asperspinidae	*Asperspina brambelli*	KF709291	JQ410991	JQ410990	no data
	Asperspina rhopalotecta	KF709292	KF709335	KF709259	no data
Microhedylidae	*Microhedyle glandulifera*	HQ168437	HQ168449	HQ168424	HQ168461
	Microhedyle remanei	KF709304	KF709344	KF709267	no data
	Parhedyle cryptophthalma	KF709308	JF828041	JF828042	JF828033
	Pontohedyle kepii	KC984290	JQ410967	JQ410966	JQ410912
	Pontohedyle wenzli	KC984294	JQ410958	JQ410957	JQ410907
	Ganitus evelinae	KF709312	JF828044	JF828045	JF828034
	Paraganitus ellynnae	HQ168436	HQ168448	HQ168423	HQ168460
